# Estimating the Effects of Awareness on Neck-Muscle Loading in Frontal Impacts with EMG and MC Sensors

**DOI:** 10.3390/s20143942

**Published:** 2020-07-15

**Authors:** Simon Krašna, Srđan Đorđević

**Affiliations:** 1 Faculty of Mechanical Engineering, University of Ljubljana, Aškerčeva Cesta 6, 1000 Ljubljana, Slovenia; 2 TMG-BMC, d.o.o., Štihova Ulica 24, 1000 Ljubljana, Slovenia; srdjand@tmg.si

**Keywords:** biomechanics, vehicle collision, sled tests, active response, muscle loads, MC sensor, EMG sensor

## Abstract

Critical traffic situations, such as vehicle collisions and emergency manoeuvres, can cause an occupant to respond with reflex and voluntary actions. These affect the occupant’s position and dynamic loading during interactions with the vehicle’s restraints, possibly compromising their protective function. Electromyography (EMG) is a commonly used method for measuring active muscle response and can also provide input parameters for computer simulations with models of the human body. The recently introduced muscle-contraction (MC) sensor is a wearable device with a piezo-resistive element for measuring the force of an indenting tip pressing against the surface of the body. The study aimed to compare how data collected simultaneously with EMG, video motion capture, and the novel MC sensor are related to neck-muscle loading. Sled tests with low-severity frontal impacts were conducted, assuming two different awareness conditions for seated volunteers. The activity of the upper trapezius muscle was measured using surface EMG and MC sensors. The neck-muscle load F was estimated from an inverse dynamics analysis of the head’s motion captured in the sagittal plane. The volunteers’ response to impact was predominantly reflexive, with significantly shorter onset latencies and more bracing observed when the volunteers were aware of the impact. Cross-correlations between the EMG and MC, EMG and F, and F and MC data were not changed significantly by the awareness conditions. The MC signal was strongly correlated (r = 0.89) with the neck-muscle loading F in the aware and unaware conditions, while the mean ΔF-MC delays were 21.0 ± 15.1 ms and 14.6 ± 12.4 ms, respectively. With the MC sensor enabling a consistent measurement-based estimation of the muscle loading, the simultaneous acquisition of EMG and MC signals improves the assessment of the reflex and voluntary responses of a vehicle’s occupant subjected to low-severity loading.

## 1. Introduction

Continuous efforts to improve traffic safety have prompted many in-depth studies of human-body dynamics during vehicle collisions. Anticipation and a braced response can effectively change the kinematics of a vehicle’s occupant and affect the injury outcome, particularly in cases of low-velocity impacts and the pre-crash manoeuvres of vehicles. Anthropometric test devices (ATDs), commonly used in occupant safety testing, have the inherent drawback of a passive-only response and are designed for medium- to high-severity impacts. Unlike physical testing with ATDs, human-body models for numerical simulations offer the possibility of incorporating an active response in a variety of dynamic conditions [1,2,3].

A vehicle occupant’s active response in a critical traffic situation can comprise reflex and voluntary muscle activation. The reflex activation due to a threat results in a startled response, characterised by rapid muscle co-contraction of the agonist and antagonist muscles, effectively stiffening the joints of the human body [1,4,5,6,7]. The voluntary activation is initiated by the occupant to adjust the posture or to brace in a critical situation [8,9,10]. Studies with volunteers subjected to conditions of low-severity frontal impacts, vehicle braking or lane-change manoeuvres observed smaller displacements of the upper body and a shorter onset of the neck-muscle activity when the volunteers were braced [11,12,13,14,15,16,17]. Significant effects of muscle activation level and timing related to the relaxed or tensed state were also found from computer simulations of the head and neck dynamics in the pre-crash phase and with frontal impact [18,19]. An assessment of the active muscle response is fundamental to the development of human-body models that will improve vehicle restraints and active collision-avoidance systems, which are able to alter the occupant’s muscle and kinematic responses [20,21].

Several methods of muscle control are used in active human-body modelling, like predefined muscle-activation curves and closed-loop control for stabilising posture or executing dynamic tasks [3,22]. Östh et al. [10,23] developed a whole-body human-body model (HBM) with feedback-controlled muscles for vehicle-braking simulations, noting that muscle-load sharing and muscle-recruitment patterns are two of the major challenges in developing closed-loop-controlled human-body models. Furthermore, the combination of feedback control for maintaining an initial posture, anticipatory control in a feedforward manner, and a reflex response in critical traffic situations need to be considered. Analysing the startle response is necessary for any assessment of the injury risk to drivers and occupants, particularly in vehicles with autonomous-manoeuvring capabilities required by legislation. The transition between the startle response and voluntary action needs to be investigated further to support the development of vehicle restraints and active safety systems [10,24,25]. Hence, there is an increasing need to study the human-body active response in autonomous-vehicle manoeuvres and low-severity impacts, where timing and the pattern of muscle activation play a crucial role.

The conventional method for providing the experimental data on active muscle response is electromyography (EMG), using surface or fine-wire electrodes to measure the muscle’s electrical activity. As a non-invasive technique, surface EMG is widely applied, but it is also subject to numerous factors in the measuring and processing of EMG data that can compromise the outcome [26,27,28,29,30]. The post-processed and normalised EMG signal represents a time-dependent input parameter for muscle-activation dynamics that introduce a delay between the muscle’s electrical and mechanical onset, and non-linear relation between the EMG and the generated muscle force [31,32]. With the activation known, a Hill-type phenomenological model [33] is commonly used for the contraction dynamics to simulate the force generated by the muscle-tendon units. The active force component depends on the muscle’s length and contraction velocity, being proportional to the activation in isometric conditions. The passive force component is related to the tissue’s elasticity and cannot be detected by EMG. In summary, relating experimental data on muscle activity to the HBM active response requires a description of the muscle activation and contraction dynamics. However, the limitations of the EMG can introduce difficulties in the modelling of mechanical muscle response in short dynamic tasks, which is crucial for understanding the vehicle-occupant dynamics in critical events [5].

Đorđević et al. [34,35] introduced a muscle-contraction (MC) sensor, which is a patch-like electronic device with an indenting tip pressed perpendicularly to the body surface. It has a piezo-resistive element that is sensitive to muscle tension that can result from active contraction or a passive tensile load. The MC signal (indentation force of the tip of the sensor) showed a nearly linear correlation (Pearson’s r = 0.976) with muscle force for isometric contractions [35]. For eccentric contractions in conditions of low-severity frontal impacts, a Pearson’s r = 0.821 was obtained between the MC signal and the estimated load of the upper trapezius muscle in our previous study [36].

The present study is focused on assessing the neck-muscle loads in simulated frontal impacts with healthy volunteers. The main interest is to identify possible differences and correlations between the data from EMG and motion capture methods, and the novel MC sensor method, in aware and unaware conditions. The first aim of the study is to analyse how the EMG, kinematic, and MC sensor data, related to the muscle active and passive loading, differ in the time domain. The second aim is to quantify the differences between the EMG, kinematic, and MC sensor data, and to assess their consistency between the two awareness conditions.

## 2. Materials and Methods

### 2.1. Experiment Setup

This study is based on the experimental data from the sled tests performed and partially described by Krašna et al. [36]. In addition, signal- and data-processing methods are revised for this study. A total of 11 healthy volunteers participated, 8 males and 3 females, age 28.0 ± 6.0 years, 178.8 ± 8.9 cm, 73.1 ± 9.4 kg. The Local Ethics Committee approved the study and before the tests, the volunteers signed informed consent. The experiments were conducted following the Declaration of Helsinki.

**Figure 1:**
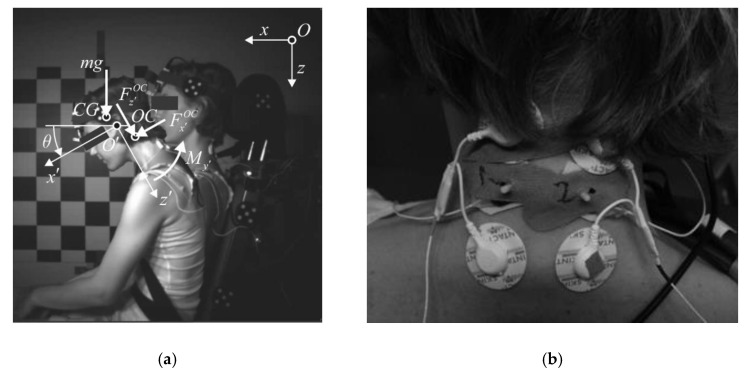
Experiment setup: (**a**) Volunteer seated on the sled from the high-speed camera point of view. The volunteer’s initial position and position during impact, and the head-neck free body diagram are depicted; (**b**) Surface EMG electrodes and MC sensors placed on the upper trapezius.

In the performed tests, the volunteers were instructed to maintain a relaxed upright posture on a rigid seat with a levelled seat base and seat back tilted at 15°, and a foot rest at 45° (Figure 1a). The volunteers were restrained with a standard 3-point seat belt. A load cell FN 4060 TI XAM-MV (FGB Instrumentation, Les Clayes-sous-Bois, France) was used to measure the tension in the shoulder-belt segment. The sled was guided with 10° inclined rails to the barrier. The sled was released from the same height to provide equal velocity for a particular impact condition. The sled deceleration was measured with a uni-axial accelerometer SD 2012–50 (Silicon Designs, Kirkland, USA).

Altogether, five impact conditions were simulated with the sled tests: low, medium, and high severity aware; low severity without foot rest; and low severity unaware. Impacts were delivered in the same order for all the volunteers, gradually increasing the dropping height of the sled and the impact severity to prevent the volunteers from possible discomfort. Two sets of low-velocity frontal impacts were completed, Δv = 7.6 ± 0.4 km/h, peak deceleration 1.7 ± 0.1 g. The first set aimed to measure the dynamic response of a volunteer aware of the impact, as partially presented in the previous study [36], and was processed again for the purpose of this study. In addition, a second measurement set, obtained during the same sled tests, is included for which the volunteers were asked to close their eyes during the pre-impact phase to simulate the dynamic response of an unaware occupant. Each volunteer performed two repeated tests for the aware and the unaware conditions with the time of 3 min between the sequential trials, a total of 44 trials in two complete sets of the measured data.

A five-point marker was attached to the side of the volunteer’s head and a 3-axis accelerometer was attached to the forehead. A high-speed camera Ultima 512 (Photron, Tokio, Japan) mounted on the sled was triggered at the instant of impact, capturing the volunteer’s sagittal-plane motion at 1000 fps. The motion of the marker was tracked with the Kinovea 0.8.15 software tool. A smoothing spline was applied to the path of the tracked marker. For the head-motion analysis, custom functions were written in Matlab 2016b (Natick, MA, USA) based on the approach presented by Challis [37].

The EMG signals were collected with Ag/AgCl surface electrodes (Skintact F-301, Innsbruck, Austria) positioned bilaterally on the upper trapezius muscle (Figure 1b) and recorded with a Biosignalplux (PLUX, Lisbon, Portugal) at a sampling rate of 1 kHz. The raw EMG signals were band-pass filtered with a Butterworth 6th-order zero-phase filter for the 10–400 Hz range. The EMG root-mean-square (RMS) value was determined with a 25-ms moving-average window and a smoothed low-pass Butterworth 6th-order zero-phase filter with 15-Hz cut-off. Two MC sensors (MC-System, TMG-BMC, Slovenia) for mechanomyography were attached bilaterally to the upper trapezius in between the EMG electrodes, while two MC sensors were placed bilaterally on the vastus medialis. The indenting tip of the MC sensor protrudes into the outer surface of a muscle, measuring the indentation force with a piezo-resistive element [34]. The MC signal, sampled at 1 kHz, was low-pass filtered with a 6th-order zero-phase Butterworth filter with a 20-Hz cut-off. The EMG and MC signals recorded from the left- and the right-hand sides were averaged for further processing. The time was set to zero at the instant of the impact. The baseline of the signals was set to the mean signal value over 0.5 s from −3.0 s to −2.5 s before the impact, when the sled had not yet started to move. The processed signals were then cut to the interval from 0.5 s before impact to 0.9 s after the impact and normalised to the peak value observed during the trials for each volunteer individually.

Time delays Δ and cross-correlation coefficients r between the EMG signal, the MC neck signal, and the neck-muscle force F were estimated pairwise with a normalised cross-correlation over the period 0.0–0.9 s. The onset of the neck-muscle activity from the instant of impact was determined by visually inspecting the EMG and MC signals measured and the neck-muscle load estimated (Section 2.2) [38,39,40]. The EMG onset latency was defined as the time of the initial burst of the band-pass-filtered EMG signal. For the MC signal and the neck-muscle load, the onset latency was defined as the instant of the positive rate’s occurrence for the processed MC signal and the neck-muscle load estimated from an inverse dynamics analysis. The onset latencies of the EMG and MC signals from the left- and right-hand sides were averaged.

### 2.2. Analysis of the Dynamic Response

Kinematic data from the motion capture were used for the planar-inverse-dynamics analysis to estimate the neck-muscle loads during the impact [13,36,41,42]. The head was considered as a rigid body connected to the neck at the occipital condyles (OC). The head’s local coordinate system x′y′z′ was defined in the midsagittal plane with the origin O′ in the external auditory meatus; the local x′-axis pointing anteriorly along the Frankfurt plane; and the z′ down and the y′ to the right-hand side; whereas the global x-axis was directed forward, horizontally; and the z-axis vertically downwards (Figure 1a). The flexion/extension angle of the neck θ was defined as the angle between the local x′ and the global x axis. The location of the occipital condyles $\left({x\prime }^{OC},{z\prime }^{OC}\right)$ and the head’s centre of gravity $\left({x\prime }^{CG},{z\prime }^{CG}\right)$ with respect to the local system origin O′ were defined as reported by Yoganandan et al. [43].

The global coordinates of the head’s motion were first determined from high-speed videos and differentiated to obtain the CG acceleration in terms of the global coordinates:

(1) $${\vec{a}}^{CG}=\vec{a}+\vec{\alpha }\times {\vec{s}}^{CG}+\vec{\omega }\times \left(\vec{\omega }\times {\vec{s}}^{CG}\right)$$

where $\vec{\omega }$ is the angular velocity, $\vec{\alpha }$ is the angular acceleration, ${\vec{s}}^{CG}$ is the position vector of CG with respect to the local system origin, and $\vec{a}$ is the translational acceleration of the head’s local system origin. Based on the measured kinematic data for the head motion, the following set of equations can be used to determine the shear force (Equation (2)), the axial force (Equation (3)), and the net bending moment (Equation (4)) for the OC joint:

(2) $$F_{x\prime }^{OC}=ma_{x\prime }^{CG}-mgsin\theta$$

(3) $$F_{z\prime }^{OC}=ma_{z\prime }^{CG}-mgcos\theta$$

(4) $$M_{y\prime }=I_{y\prime }^{O\prime }\alpha -F_{x^{\prime }}^{OC}{z^{\prime }}^{OC}-F_{z\prime }^{OC}{x\prime }^{OC}-mgsin\theta {z\prime }^{CG}-mgcos\theta {x\prime }^{CG}$$

where $a_{x\prime }^{CG}$ and $a_{x\prime }^{CG}$ are components of the CG acceleration (Equation (1)) in terms of the head’s local coordinates, and g is the acceleration due to gravity. The head’s mass m and the moment of inertia $I_{y\prime }^{O\prime }$ for the local $y^{\prime }$-axis was defined for the male and female volunteers from Yoganandan et al. [43]. For the neck flexion resulting from the frontal impacts analysed in our study, it was assumed that the upper trapezius muscle generated the neck extension moment, maintaining a dynamic equilibrium around the OC joint. Based on the study by Anderst et al. [44], the upper trapezius moment arm was defined as linearly dependent on the flexion angle θ, which together with the moment (Equation (4)), made it possible to estimate the time history of the equilibrium load F generated by the neck’s extensor muscles.

### 2.3. Statistical Analysis

The data collected were first analysed for peak values, the timing of the peak values, and onset latencies. After being checked for normality with the Shapiro-Wilk test [45], the data on the peak values and the timing of the peak values were compared for the difference between the aware and the unaware trials. The normally distributed parameters were compared with a paired t-test. For the non-normal parameters, we used the non-parametric Wilcoxon signed rank test.

Two-way repeated measures ANOVAs (3 × 2 design) were performed to test the differences between the time delays (ΔEMG-MC, ΔEMG-F, ΔF-MC) and between the cross-correlation coefficients (rEMG-MC, rEMG-F, rF-MC) in the aware and unaware conditions. Shapiro–Wilk test for normality, Grabb’s test for detecting outliers, and Mauchly’s test for sphericity were used. The significance threshold was set to 0.05.

## 3. Results

**Figure 2:**
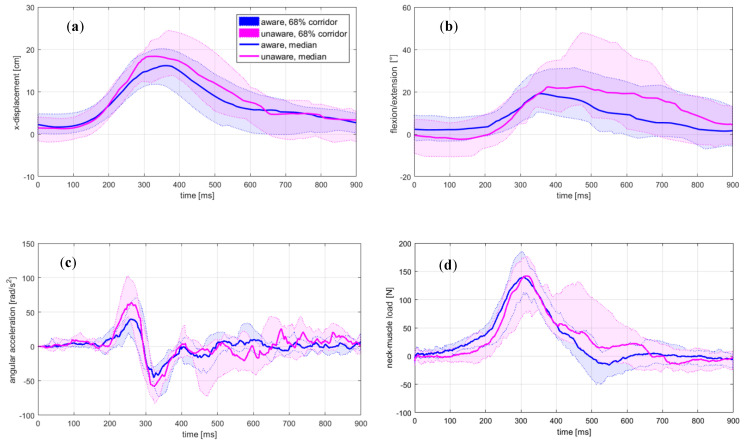
Head-neck’s motion from motion-capture and inverse dynamics analyses: (**a**) horizontal excursion, (**b**) flexion, (**c**) angular acceleration; (**d**) estimated load for the neck’s extensor muscles.

**Figure 3:**
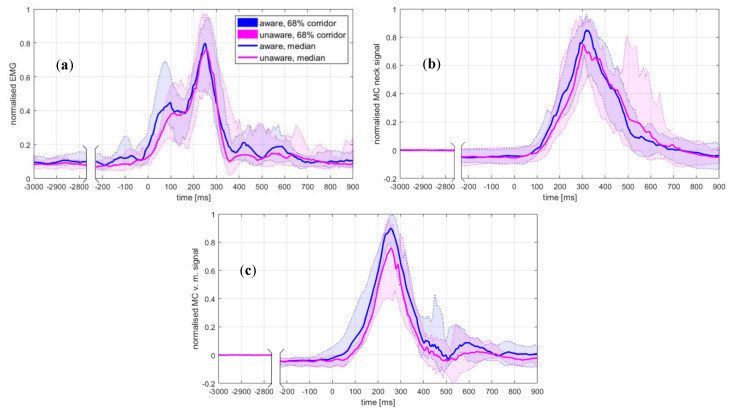
(**a**) EMG linear envelope for the upper trapezius, (**b**) muscle-contraction (MC) sensor signal for the upper trapezius, (**c**) MC signals for vastus medialis. Note that the median values of the MC signals (**b**,**c**) before the impact are below the baseline estimated for the interval −3.0 to −2.5 s, due to the gravity component acting on the sled’s downwards motion along the 10° inclined rails, effectively reducing the muscle force needed to maintain the volunteer’s initial posture before the impact.

The time histories of the head-motion parameters, EMG, and MC signals are presented in Figure 2 and Figure 3. The results for the peak values are summarised in Table 1 and Table 2.

The unaware condition resulted in significantly increased magnitudes of the horizontal head excursion, head-neck flexion, and angular acceleration. At the moment of impact, the head was positioned anteriorly and rotated forwards in the aware trials. The magnitudes of the OC loads remained at the same level, while the seatbelt force was higher for the unaware condition. The peak values of the analysed quantities consistently occurred later in the unaware trials, but statistically, a significant change was observed only for the head-neck’s flexion. The curve shape for the neck-muscle load F (Figure 2d) and the MC neck signal (Figure 3b) indicates an increased neck extensor tension in unaware trials at about 400–600 ms, not observed in the EMG curve (Figure 3a). The peak value for MC v. medialis was significantly lower in the unaware trials, but occurred at approximately the same instance (Figure 3c).

**Figure 4:**
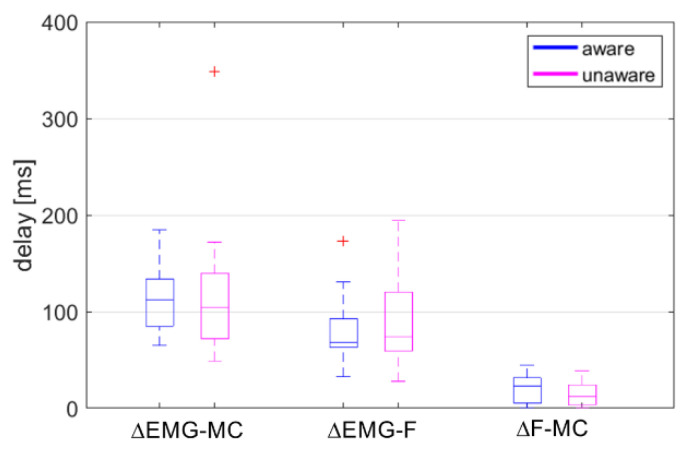
Time delays estimated with cross-correlation between EMG, MC neck, and neck-muscle load F. Positive sign of Δ indicates that the MC neck is delayed relative to the EMG (ΔEMG-MC), the F is delayed relative to the EMG (ΔEMG-F), and the MC neck is delayed relative to F (ΔF-MC). Depicted: median value, 25th- and 75th-percentile values, min-max range and outliers (red), aware (blue), unaware (magenta).

Visual inspection of the results revealed an additional EMG peak at 600–700 ms for the second unaware trial of Subject 03 (female), contributing to the low cross-correlation coefficient for the trial (0.4335 for rEMG-F) and the estimated delay ΔEMG-F of -339 ms, together with outliers detected for the ΔF-MC in both awareness conditions. Therefore, the data from Subject 03 were dropped from further analyses of the cross-correlations and time delays (Table 3, Figure 4).

The normality of the three groups of delays ΔEMG-F, ΔEMG-MC and ΔF-MC for the aware and unaware conditions was checked with the Shapiro-Wilk test, indicating a non-normal distribution of the ΔEMG-F for the aware condition (*p* = 0.022) and the ΔEMG-MC for the unaware condition (*p* < 0.001). After removing two outliers detected with the Grabb’s test, the normal distribution assumption was not rejected. Removing the outliers did not influence the conclusions based on two-way repeated measures ANOVA. Therefore, the results of the analysis on the whole dataset are reported. A significant main effect was found for the groups of delays (F(2,38) = 54.39, *p* < 0.001, $\eta_{p}^{2}$ = 0.74), but no main effect of awareness (F(1,19) = 0.02, *p* = 0.891, $\eta_{p}^{2}$ < 0.01) or interaction effect between the groups of delays and awareness (F(1.50,28.54) = 0.77, *p* = 0.437, $\eta_{p}^{2}$ = 0.04). Bonferroni post-hoc tests showed significant differences between all three groups (*p* < 0.001), with ΔF-MC = 17.8 ± 14.1 ms, ΔEMG-F = 83.4 ± 39.0 ms, and ΔEMG-MC = 114.4 ± 53.3 ms. The ΔF-MC showed shorter delay and less scatter in both the aware and unaware trials (Figure 4). Overall, the differences between the aware and unaware trials were small for all three groups.

The time-history profiles for the EMG, MC neck and neck-muscle force F were compared based on their cross-correlation, specifically the cross-correlation coefficients rEMG-MC, rEMG-F and rF-MC (Table 3). The assumption of normality was violated only for rEMG-MC in unaware trials (*p* = 0.019). A two-way repeated measures ANOVA showed a significant difference in the cross-correlation coefficients between the rEMG-MC, rEMG-F and rF-MC (F(2,38) = 28.01, *p* < 0.001, $\eta_{p}^{2}$ = 0.60), but no effect of the awareness conditions (F(1,19) < 0.01, *p* = 0.981, $\eta_{p}^{2}$ < 0.01) or the interaction between the two factors (F(1.50,28.49) = 0.22, *p* = 0.743, $\eta_{p}^{2}$ = 0.01). Post-hoc tests revealed that the rF-MC was significantly bigger than the rEMG-MC and rEMG-F for both awareness conditions (*p* < 0.001). The cross-correlation coefficients indicate that the neck-muscle load F (Figure 2d) was more closely correlated with the MC signal than with the EMG signal (Figure 3a,b).

**Figure 5:**
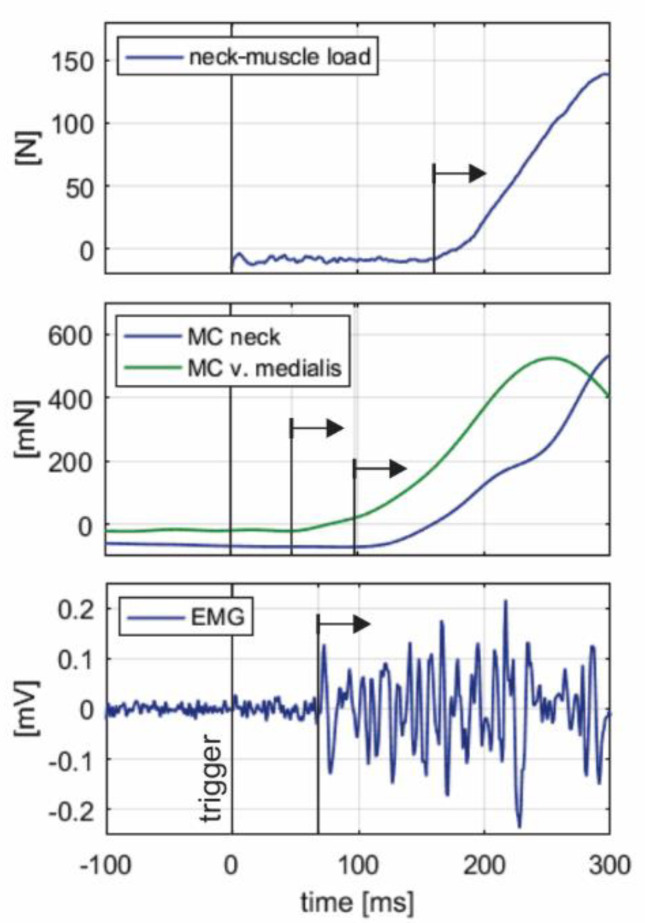
An example of onset latencies for the neck-muscle load F, MC neck and MC v. medialis (indentation force of the tip of the sensor), and EMG signals from a single trial. Black arrows indicate visually determined onset for each of the signals collected. Note that F was estimated based on inverse dynamics analysis of the head-neck motion from the instant of impact (trigger).

Figure 5 shows representative initial segments of neck-muscle load F, MC neck and MC v. medialis signals, and EMG signal, collected in a single trial, with visually determined onset latencies.

The onset latencies for EMG, MC neck and MC v. medialis were significantly longer for the unaware trials (Table 4). The onset of MC v. medialis occurred before the impact in the first unaware trials of Subjects 05, 07, 09. The onset latency for the estimated neck-muscle load F was not changed significantly (*p* = 0.060), although it was longer for the unaware trials.

A few subjects exhibited an initial increase of the neck-muscle load, followed by a local decline at 150–250 ms. Further analyses showed that a combination of axial and shear loading might occur, which reduced the equilibrium bending moment and neck-muscle load according to Equations (2)–(4). Hence, the onset of the neck-muscle load could be detected at a later instant, and no specific indications were found for individual subjects and awareness conditions. The shear force also had a dominant contribution in a steady increase of the neck-muscle load observed in some of the trials from the instant of impact to approx. 200 ms, which indicates the neck extensor effort to stabilise the upright head position before the neck flexion was initiated.

## 4. Discussion

Experimental data on the head-neck muscle activity and kinematics from 11 healthy volunteers were collected in a series of sled tests simulating low-severity frontal impacts of a vehicle, where two conditions for the volunteers’ awareness of an impending impact were compared. The main finding of the study is that data simultaneously recorded in the same area of trapezius muscle with EMG and MC sensors are different. The results suggest that analysis of motion capture, EMG, and MC data combined can represent a basis for assessing the muscle activation and loading under different awareness conditions.

Inverse dynamics analysis and the time domain analysis of the EMG and the MC sensor signals from the upper trapezius showed a significantly smaller delay and a higher cross-correlation between the estimated neck-muscle load F and the MC signal than between EMG and F, and between EMG and MC. The same tendency of differences between F and MC, EMG and F, and EMG and MC is observed for the aware and the unaware conditions. Both the MC and EMG signals showed significantly longer onset latencies for the unaware conditions.

The dynamic response of the volunteers was comparable to the previous studies of low-severity frontal impacts. The mean head excursion was significantly influenced by the awareness and was decreased for the aware trials to 140.8 ± 38 mm (Table 1), compared to 168 ± 35 mm at the peak deceleration of the sled (2.6 g) in the previous study [36]. Beeman et al. [11,12,13] observed a 124 ± 9 mm head excursion at 2.5 g for relaxed volunteers in the driver’s position with the arms acting against the steering wheel. Ólafsdóttir et al. [20] found a similarly reduced head excursion contributed to the reflexive response evoked by the belt pre-tensioner. Seatbelt force was significantly reduced and the peak values for MC v. medialis were higher in the aware trials (Table 1), indicating the effect of the lower extremities pushing the body against the seat back and contributing to a more controlled upper-body motion, particularly in an occupant position with no arms engaged [11,16,46]. A similar behaviour was also observed in the unaware trials of Subject 11, resulting in a very low peak value of the seatbelt force (47.2 N, Table 1).

The kinematic response concerning the state of awareness was comparable to the study by Ejima et al. [15,16], despite the differences in the restraining and positioning of the volunteers. Peak values and timings for the peak OC loads were not significantly different between the aware and the unaware condition (Table 1 and Table 2). A similar situation was observed by Beeman et al. [13], when comparing OC loads for a relaxed and braced response. The estimated peak timing of the bending moment and the neck-muscle load remained close in both conditions (Table 2).

The inverse-dynamics analysis of the neck loads was based on a simple model assuming that the upper trapezius provides most of the net moment needed for dynamic equilibrium around the head-neck joint, which is in accordance with the findings of several other studies. In a similar setup, only minor activity of the neck flexors was observed for relaxed volunteers [16], which can likely be explained by the mechanism of reciprocal inhibition [47]. The osteo-ligamentous stiffness of the cervical spine is known to be low on the physiological range of motion, comparing to the contribution of passive and active muscle to the equilibrium OC moment [48,49]. The head-neck posture is mainly stabilised by deep neck muscles, while superficial neck muscles are activated during dynamic tasks, providing more mechanical advantage and a faster response [14,50,51,52,53]. A computational study by Hedenstierna et al. [28] identified trapezius and splenius capitis as dominant in response to frontal impacts. The head-neck model used cannot take into account detailed inter-segmental kinematics and load distribution over several muscles with different moment arms [54]. Rather, it provides an estimation of the cumulative neck-muscle load that can be related to the EMG and MC signals measured at the upper trapezius. The MC sensor attached to the muscle surface also detects the passive tension due to the muscle wrapping during motion. Further, despite the fact that normalisation to the maximum voluntary isometric contraction is preferred in modelling the active muscle response, obtaining the maximum values of EMG and MC signals might be hard in the case of trapezius muscles [30], making normalisation to the peak values observed during the trials a viable alternative to compare the magnitudes of the signals.

The volunteers exhibited significantly different timings of the active response (Table 4). The awareness conditions for the volunteers were defined in a simple manner in our study, compared to the effort put into controlling the visual, auditory and vestibular stimuli when analysing the effect of anticipation, using visual and audible means of distractions before the impact [11,55]. However, in the studies in which the subjects were instructed to perform simple physical tasks with their eyes open or closed, the volunteers showed different muscle responses between the two awareness conditions [6,56,57].

The timing of the peak values of the EMG, MC neck, MC v. medialis, and the neck-muscle force was not changed significantly with awareness (Table 1), while the onset consistently occurred later for the unaware conditions (Table 4), implying a steeper rise from the onset to the peak for the EMG, MC neck, and neck-muscle load F. This is in agreement with results of Kumar et al. [14], who observed a steeper slope for the upper trapezius EMG profile in conditions of an unexpected frontal impact. Although Kumar et al. [14] found that the time to peak EMG for trapezius was significantly affected by the expectation of the impact across the range 0.5–1.4 g of sled acceleration, they observed only minor differences for 1.4 g. For a few of the volunteers, the load F onset latency estimated was longer in the aware trials. A possible reason for this was that those volunteers, following the instructions to relax, were consciously delaying active response in the aware trials. The fastest response was observed for the MC v. medialis signal (Figure 3c), which had a significantly higher peak value (Table 1) and a shorter onset latency (Table 4) for the aware condition, although the peak-value timing was not changed. A large minimum to a maximum range of the MC v. medialis onset latencies in the aware trials was due to the pre-impact activity of the lower extremities observed for Subjects 10 and 11. The offset between the rise segments of the aware and unaware curves (Figure 3c) can probably be attributed to the synchronous and higher recruitment of vastus medialis muscle fibres. The seat-belt force was significantly larger in the unaware trials (Table 1), which implies that the effort of the lower extremities and lumbar in stabilising the body posture was higher with a visual clue of the impending impact that was present in the aware trials.

The initial burst of the median EMG profile at 0–150 ms (Figure 3a) was not transferred to an increase of the estimated muscle force (Figure 2d), neither could it be observed on the MC neck signal (Figure 3b), regardless of the awareness condition. Based on the video recordings, no indications of a motion artefact were found. A further inspection of the EMG signals showed a greater tendency for the burst to appear in the aware trials. The peak of the burst appeared at 80.1 ± 19.7 ms in the aware trials and 109.2 ± 23.0 ms in the unaware trials (p = 0.003), while the burst decay to the local minimum lasted 65.8 ± 23.1 ms and 61.7 ± 18.9 ms, respectively. Moreover, the onset of the EMG and MC signals preceded the onset of the neck-muscle load F (Table 4). Given that F was estimated from an inverse-dynamics analysis, according to Equations (2)–(4), the onset of F needed to maintain the dynamic equilibrium occurred only after the head motion was initiated at approx.100–200 ms after the impact (Figure 2). Hence, for both awareness conditions, the initial EMG burst can be related to the reflexive activation evoked by the visual and vestibular stimuli at the impact [1,6]. The burst was more noticeable in the aware trials (Table 4), which were conducted as the first set of conditions in the study. Although the attenuation of the burst in the unaware trials could be partially attributed to the habituation of the volunteers, as observed in previous studies comparing first trial responses with subsequent ones [7,58], the overall dynamic response showed that the volunteers were more startled in the aware conditions.

The delays between the EMG, MC and neck-muscle load F were estimated with a cross-correlation, as used in several studies [59,60,61]. A cross-correlation is sensitive to the timing and the curve profile, also giving an estimate of the curve similarity and being recognised as an appropriate method for evaluating the signal-peak timing and the peak-to-peak differences between signals [62,63]. The cross-correlation estimated delays ΔEMG-MC, ΔEMG-F and ΔF-MC were not changed significantly by the awareness conditions, except for a small difference in the ΔF-MC delay, being significantly shorter and with a smaller variance than the ΔEMG-F delay (Figure 4). The mean coefficient of cross-correlation rF-MC was significantly higher in both the aware and unaware trials, r = 0.89–0.90, versus r = 0.80–0.81 for rEMG-F (Table 3). The MC neck- and muscle-load profiles exhibited a 68-percentile corridor wider at 400–600 ms for the unaware trials, after the peak values at 342.1 ± 66.0 ms and 351.6 ± 90.7 ms (Table 2). Since the cross-correlation coefficients for rF-MC were consistently high for both conditions representing different levels of startle response, it is possible to conclude that the MC signal remained closely related to the time profile and the magnitude of the neck-muscle load, regardless of response variations between the volunteers. Higher peak values in the unaware conditions were observed for the head-neck’s flexion (Figure 2b) and the MC neck signal (Figure 3b), but not for the EMG signal (Figure 3a), suggesting that the MC signal is partially related to passive muscle tension during neck flexion.

The results presented show that EMG, being directly related to neural excitation [64,65], detects the earliest sign of muscle reflex and voluntary activity and is correlated with the state of awareness. However, using the EMG signal as a muscle-activation parameter requires a series of decisions on processing the raw signal and modelling the muscle-activation and contraction dynamics, besides the other parameters of the musculoskeletal system, to finally estimate the muscle contribution to the joint moment generated [31,33,66]. Comparing to EMG, the better agreement between MC signal and muscle loading was found after a straightforward measuring and processing procedure. In addition to EMG, the MC signal represents a set of experimental data on muscle loading that can be used when analysing and modelling the reflex or voluntary response.

The study was limited to analysis of experimental data with the help of a simple planar model to estimate the neck-muscle loading based on inverse dynamics analysis. Future study should involve larger sample size and carefully controlled awareness conditions. Due to the complexity of the human body, extending data collection to 3D motion of multiple body segments, external loads, and activity of antagonistic muscle pairs would provide more insight on the dynamic response of the volunteers. With advanced human body modelling, a complete set of anatomical structures and muscles of the spine region could be analysed for their contribution to inter-segmental loading and motion.

## 5. Conclusions

Understanding the active human response is essential for modelling the reflex and anticipatory behaviour of vehicle occupants during low-severity impacts and accident-avoiding manoeuvres. Sled tests with healthy volunteers were performed, simulating low-severity frontal impacts to compare the motion of the head-neck complex and muscle activity with respect to an awareness of the impending impact. The volunteers’ kinematic responses were comparable with those of other studies of low-severity frontal impacts, showing larger magnitudes of head-neck motion in the unaware trials. EMG and MC signals measured on the neck extensor muscles yielded a significantly later onset in the unaware trials. An EMG burst indicating a reflex response was observed, while the MC signal was closely correlated with the estimated neck-muscle load. The results of the study showed that the simultaneous acquisition of MC signals, in addition to EMG, provides complementary data on the active and passive loading of the muscle-tendon complex. Furthermore, it improves the experimental assessment of the reflex and the anticipatory response of a vehicle occupant when exposed to conditions of low-severity impacts or the emergency manoeuvres of vehicles.

## Figures and Tables

**Table 1:** Peak values for the head-neck motion, neck loads, normalised EMG, and normalised MC sensor signals in the aware and unaware trials: mean ± standard deviation (SD), median (min/max), and corresponding *p*-values for pairwise comparisons between the aware and unaware conditions.

Peak Values	Aware		Unaware		*p*-Value
	Mean ± SD	Median (min/max)	Mean ± SD	Median (min/max)	
**head excursion [mm]**	140.8 ± 38.0	130.4 (89.5/209.1)	182.6 ± 71.0	166.9 (71.5/382.2)	**0.049** ^a^
head-neck flexion [°]	18.3 ± 9.6	16.8 (3.2/41.0)	30.2 ± 17.1	25.3 (9.7/69.0)	**0.001** ^a^
head acc. [m/s^2^]	32.6 ± 2.7	32.3 (28.3/38.9)	33.3 ± 3.7	34.1 (26.1/39.2)	0.412
angular acc. [rad/s^2^]	−51.5 ± 22.9	−48.8 (−103.0/−10.7)	−64.0 ± 20.0	−61.6 (−114.2/−21.4)	**0.028**
axial force [N]	13.0 ± 12.3	12.6 (−17.7/33.5)	10.8 ± 14.8	13.8 (−37.2/25.9)	0.307 ^a^
shear force [N]	103.9 ± 12.1	105.0 (80.5/130.9)	105.4 ± 16.3	106.9 (77.1/131.6)	0.682
bending moment [Nm]	−6.37 ± 1.02	−6.21 (−8.26/−4.21)	−6.40 ± 1.20	−6.44 (−8.53/−4.17)	0.808 ^a^
neck-muscle load [N]	149.6 ± 29.4	144.6 (95.2/207.6)	157.4 ± 34.5	149.6 (99.9/237.7)	0.258
seatbelt force [N]	725.3 ± 316.0	768.2 (263.4/1572)	869.3 ± 400.6	897.0 (47.2/1521)	**0.030**
EMG neck [−]	0.791 ± 0.181	0.807 (0.469/1)	0.849 ± 0.136	0.843 (0.613/1)	0.211 ^a^
MC neck [−]	0.878 ± 0.128	0.891 (0.508/1)	0.874 ± 0.136	0.919 (0.546/1)	0.685 ^a^
MC v. medialis [−]	0.901 ± 0.100	0.899 (0.676/1)	0.772 ± 0.203	0.797 (0.347/1)	**0.031** ^a^
head init. position *x* [mm]	22.0 ± 25.5	22.4 (−36.8/65.4)	12.1 ± 25.5	14.4 (−36.8/52.9)	**0.022**
head-neck init. flexion [°]	2.9 ± 5.4	2.3 (−3.4/10.0)	−1.3 ± 8.1	−0.5 (−18.8/12.4)	**0.015**

^a^ non-normal distribution; bold faced values indicate statistical significance (*p* < 0.05).

**Table 2:** Timing of peak values for the head-neck motion, neck loads, EMG, and MC signals for the aware and unaware trials [ms]: mean ± SD, median (min/max), and corresponding *p*-values for pairwise comparisons between the aware and unaware conditions.

Timing [ms]	Aware		Unaware		*p*-Value
	Mean ± SD	Median (min/max)	Mean ± SD	Median (min/max)	
**head excursion**	331.2 ± 26.4	327.5 (292/401)	351.3 ± 47.1	339.5 (269/468)	0.095
head-neck flexion	389.2 ± 40.0	379.5 (337/472)	457.1 ± 88.2	451.0 (334/584)	**0.007**
head acc.	288.0 ± 15.9	287.0 (265/316)	294.4 ± 45.5	282.0 (241/412)	0.876
angular acc.	327.3 ± 19.5	330.5 (291/363)	331.8 ± 41.9	333.5 (235/424)	0.670
axial force	323.4 ± 82.2	283.5 (227/477)	188.6 ± 46.8	174.5 (152/340)	0.079 ^a^
shear force	291.7 ± 19.1	291.5 (255/337)	301.1 ± 46.0	285.5 (241/426)	0.615 ^a^
bending moment	307.3 ± 17.6	304.5 (283/357)	333.5 ± 60.2	313.5 (252/484)	0.081
neck-muscle load	310.2 ± 17.0	306.5 (288/357)	342.1 ± 66.0	315.5 (252/484)	0.095 ^a^
seatbelt force	259.7 ± 16.5	261.1 (230/301)	256.3 ± 36.4	250.0 (213.9/370)	0.183 ^a^
EMG	232.0 ± 53.4	243.0 (73/281)	266.5 ± 87.4	251.0 (200/457)	0.284 ^a^
MC neck	319.6 ± 39.6	310.5 (271/449)	351.6 ± 90.7	327.0 (259/601)	0.263 ^a^
MC v. medialis	262.2 ± 22.3	259.0 (233/328)	259.3 ± 21.3	254.5 (228/318)	0.304 ^a^

^a^ non-normal distribution; bold faced values indicate statistical significance (*p* < 0.05).

**Table 3:** Maximum values of coefficients r estimated with cross-correlation between EMG, MC neck and neck-muscle load F; mean ± SD and median (min/max).

	Condition	Mean ± SD	Median (min/max)
rEMG-MC	aware	0.78 ± 0.09	0.78 (0.56/0.92)
	unaware	0.77 ± 0.12 ^a^	0.82 (0.54/0.91)
rEMG-F	aware	0.80 ± 0.10	0.80 (0.52/0.96)
	unaware	0.81 ± 0.09	0.81 (0.60/0.95)
rF-MC	aware	0.89 ± 0.07	0.89 (0.71/0.97)
	unaware	0.89 ± 0.06	0.90 (0.77/0.98)

^a^ non-normal distribution.

**Table 4:** Onset latencies for EMG, MC neck, MC vastus medialis, and neck-muscle load F from the instant of the impact [ms]; mean ± SD, median (min/max), *p*-value for pairwise comparisons between aware and unaware conditions.

	Condition	Mean ± SD	Median (min/max)	*p*-Value
EMG neck	aware	42.5 ± 38.2	55.5 (−77/93)	**0.001** ^a^
	unaware	91.1 ± 57.3	78.3 (2/230)	
MC neck	aware	62.8 ± 39.1	67.0 (−76/104)	**<0.001** ^a^
	unaware	124.9 ± 55.9	105.5 (55/263)	
MC v. medialis	aware	−36.0 ± 112.6	−13.0 (−352/52)	**0.003**
	unaware	36.1 ± 42.0	48.0 (−85/83)	
F neck	aware	142.5 ± 54.0	148.0 (17/224)	0.060
	unaware	180.5 ± 45.6	181.5 (79/244)	

^a^ non-normal distribution; bold faced values indicate statistical significance (*p* < 0.05).
